# Supplementary material for: The Effect of Strict State Measures on the Epidemiologic Curve of COVID-19 Infection in the Context of a Developing Country: A Simulation from Jordan
Source: Int J Environ Res Public Health. 2020 Sep 8;17(18):6530. doi: 10.3390/ijerph17186530 (PMC7558493; doi:10.3390/ijerph17186530)
Supplement: Supplementary file 1 [file ijerph-17-06530-s001.pdf]

Supplementary Table S1: Model Parameters' Description and Values Used for Simulating the Number of COVID-19 cases in Jordan under the hypothetical scenario of no-action (S2).

**Parameter and Symbols**	**Description**	**Scenario 2 Values**
β (beta)	Describes the transmission rate.	0.37
A (alpha)	Reduction in transmission rate (moderate to severe).	0.5
ε	The incubation period from the state of exposure to the disease to become infectious	1/5.2 days
P_s	Probability of developing severe SAR-CoV-2 symptoms	0.01
μ	Recovery rate	1/14 days
R_0[#]	Basic Reproduction number	5.6

[#] The R_0 formula that was used in our model is:

$\beta = R_0 / \mu + \varepsilon$

where μ is the recovery rate and ε is the rate of incubation period.

Supplementary Figure S1: Simulated COVID-19 epidemic curves in Jordan under scenarios 1 and 2 (S1 and S2), utilizing the (A) mild, and (B) Moderate to severe compartmental states.

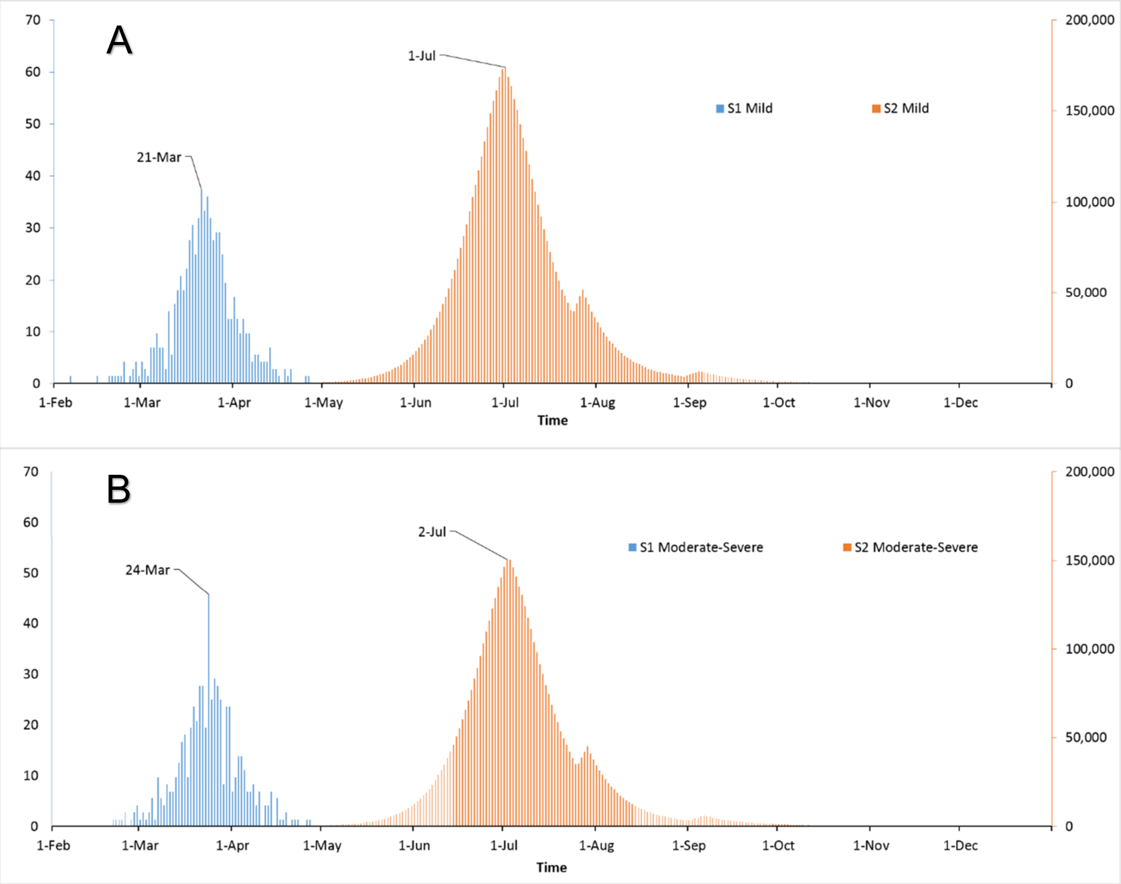

Description of Data: The simulated daily new mild COVID-19 cases under S1 peaked, on March 21, at 36 cases and a total duration of 49 days. After which, the simulated daily new mild case count started to decrease and reached, on April 27, zero daily new cases (total duration of the epidemic curve was 87 days). Estimated cumulative mild case count has reached its maximum at 794 case around April 27. Under S2, the curve peaked at 174,082 cases around July 1st (a total of 151 days). The simulated daily new moderate to severe cases (S1) reached a maximum number on March 24 with a total of 46 case (a total of 53 days). The number of simulated daily new moderate to severe

cases then decrease to zero case on April 27 (total number of days for the epidemic was 87 days). Under S2, the curve peaked at a simulated daily new cases of 150,523 on July 3 (a total of 153 days).
